# Supplementary material for: Characterization of the Effect of a Novel Production Technique for ‘Not from Concentrate’ Pear and Apple Juices on the Composition of Phenolic Compounds
Source: Plants (Basel). 2023 Sep 26;12(19):3397. doi: 10.3390/plants12193397 (PMC10574464; doi:10.3390/plants12193397)
Supplement: Supplementary file 1 [file plants-12-03397-s001.zip › plants-2496032-supplementary.pdf]

Characterization of the Effect of a Novel Production Technique for 'Not from Concentrate' Pear and Apple Juices on the Composition of Phenolic Compounds Composition

José Carlos Teixeira [1], Catarina Ribeiro [1], Rodolfo Simões [1], Maria João Alegria [2], Nuno Mateus [1], Victor de Freitas [1], Rosa Pérez-Gregorio [1,3,4*] and Susana Soares [1]

[1] LAQV-REQUIMTE. Chemistry and Biochemistry Department. Faculty of Sciences. University of Porto. Rua do Campo Alegre, 689, 4169-007, Porto, Portugal ; josecarlos_1997@hotmail.com (J.C.T.); cata-rinaribeiro23@gmail.com (C.R.); rodolfodinisimoes@hotmail.com (R.S.); nbmateus@fc.up.pt (N.M.); vfrei-tas@fc.up.pt (V.F.); susana.soares@fc.up.pt (S.S.)

[2] SUMOL+COMPAL Marcas S.A, Estr. Portela 24, 2790-179 Carnaxide, Portugal; ma-ria.alegria @sumolcompal.pt

[3] University of Vigo. Food and Agroecology Institute . Campus As Lagoas, 32004 Ourense, Spain

[4] Galicia Sur Health Research Institute (IISGS) . Department of Chemistry and Biochemistry. Food and Health Omics Group. SERGAS-UVIGO

* **Correspondence:** maria.gregorio@fc.up.pt

SUPPLEMENTARY INFORMATION

The results obtained after the tentatively identification of bounded phenolic compounds is herein listed:

Table S1- Compounds non-identified from non-extractable extraction of apple

No.	RT (min)	Ionization (ESI+/ESI-)	Molecular weight	MS/MS Product Ions
1	4.35	[M–H] +	232.93	84, 102, 158
2	5.27	[M–H] +	337.04	56, 98, 128
3	6.35	[M–H] +	182.08	91, 95, 123
4	6.59	[M–H] +	275.01	174, 193, 56
5	6.92	[M–H] +	352.33	77, 93, 75
7	7.28	[M–H] +	217.1	95, 105, 217
8	8.16	[M–H] +	214.09	95, 105
9	8.78	[M–H] +	261.13	261, 84, 56
10	9.67	[M–H] +	227.18	96, 114, 69
11	10.24	[M–H] +	305.15	56, 305, 189
12	10.61	[M–H] +	331.07	151, 141, 185
13	11.49	[M–H] +	316.21	99, 115, 159
14	11.66	[M–H] +	349.18	105, 84, 95
15	12.98	[M–H] +	393.21	393, 227, 56
16	13.13	[M–H] +	195.09	138, 110, 195
17	13.59	[M–H] +	316.21	102, 81
18	13.74	[M–H] +	340.26	114, 96, 69
19	14.18	[M–H] +	437.24	98, 227, 157

20	14.73	[M–H] +	214.09	95, 105, 77
21	15.3	[M–H] +	476.31	89, 475
22	16.3	[M–H] +	453.34	114, 96
23	18.52	[M–H] +	334.18	120, 81
24	19.02	[M–H] +	475.33	475, 227, 476
25	20.82	[M–H] +	566.43	114, 96
26	21.36	[M–H] +	588.41	588, 589
27	21.71	[M–H] +	283.71	114, 96, 69
28	26.13	[M–H] +	340.26	114, 96
29	31.49	[M–H] +	396.8	114, 96, 209
30	34.64	[M–H] +	415.16	227, 245, 209
31	37.2	[M–H] +	453.34	114, 96
32	38.94	[M–H] +	701.5	701, 702, 683
33	41.77	[M–H] +	347.24	55, 69, 93
34	42.75	[M–H] +	509.8	209, 228, 227
35	44.08	[M–H] +	679.51	209, 228, 227

Table S2- Compounds non-identified from non-extractable extraction of pear

No.	RT (min)	Ionization (ESI+/ESI-)	Molecular weight	MS/MS Product Ions
1	4.5	[M–H] +	125.98	84, 102
2	4.51	[M–H]–	304.91	174, 146
3	4.93	[M–H]–	197.8	162, 197, 160
4	5.1	[M–H]–	215.03	89, 113, 71
5	5.32	[M–H]–	377.08	341, 377, 342
7	5.32	[M–H]–	387.11	341, 342, 269
8	5.43	[M–H] +	365.1	365, 366
9	7.09	[M–H] +	352.33	75, 59, 93
10	7.09	[M–H]–	112.99	69,112, 70
11	7.09	[M–H] +	141.96	113, 131, 72
12	15.99	[M–H] +	261.13	161, 262, 202
13	16.24	[M–H] +	227.17	227, 228
14	18.43	[M–H] +	305.15	305, 306
15	18.49	[M–H] +	245.05	183, 215, 167
16	18.49	[M–H] +	215.04	183, 155, 119
17	18.85	[M–H] +	114.09	114, 115
18	20.47	[M–H]–	137.02	137,138, 92
19	20.47	[M–H]–	181.07	89, 92, 101
20	23.2	[M–H] +	362.24	362, 363
21	23.71	[M–H] +	295.11	202, 203, 295
22	24.72	[M–H] +	250.17	250, 158, 91
23	25.49	[M–H] +	209.08	177, 209, 149
24	27.13	[M–H] +	475.32	475, 476
25	27.15	[M–H] +	453.34	435, 322, 228
26	29.31	[M–H]–	347.11	319, 315, 259
27	29.61	[M–H] +	588.4	588, 589
28	30.09	[M–H] +	245.05	183, 200, 167, 119
29	31.36	[M–H] +	701.49	701, 702, 203
30	32.25	[M–H] +	278.21	278, 91, 186

31	32.54	[M–H] +	814.57	814, 815, 203
32	34.19	[M–H] +	209.08	55, 103, 149, 177, 209
33	34.26	[M–H]–	187.09	125, 126, 169, 187
34	39.2	[M–H] +	401.15	330, 315, 167
35	40.38	[M–H] +	387.14	337, 305, 277, 167
